# Purification and Characterization of Ornithine Decarboxylase from *Aspergillus terreus*; Kinetics of Inhibition by Various Inhibitors

**DOI:** 10.3390/molecules24152756

**Published:** 2019-07-29

**Authors:** Ashraf S.A. El-Sayed, Nelly M. George, Marwa A. Yassin, Bothaina A. Alaidaroos, Ahmed A. Bolbol, Marwa S. Mohamed, Amgad M. Rady, Safa W. Aziz, Rawia A. Zayed, Mahmoud Z. Sitohy

**Affiliations:** 1 Enzymology and Fungal Biotechnology Lab (EFBL), Botany and Microbiology Department, Faculty of Science, Zagazig University, Zagazig 44519, Egypt; 2 Biology Department, Faculty of Science, King Abdulaziz University, Jeddah 21955,Saudi Arabia; 3 Faculty of Biotechnology, Modern Science and Arts University, Cairo 12566, Egypt; 4 Department of Laboratory and Clinical Science, College of Pharmacy, University of Babylon, Babylon 51002, Iraq; 5 Pharmacognosy Department, Faculty of Pharmacy, Zagazig University, Zagazig 44519, Egypt; 6 Biochemistry Department, Faculty of Agriculture, Zagazig University, Zagazig 44519, Egypt

**Keywords:** *Aspergillus terreus*, curcumin, ornithine decarboxylase, kinetics, inhibition

## Abstract

l-Ornithine decarboxylase (ODC) is the rate-limiting enzyme of *de novo* polyamine synthesis in humans and fungi. Elevated levels of polyamine by over-induction of ODC activity in response to tumor-promoting factors has been frequently reported. Since ODC from fungi and human have the same molecular properties and regulatory mechanisms, thus, fungal ODC has been used as model enzyme in the preliminary studies. Thus, the aim of this work was to purify ODC from fungi, and assess its kinetics of inhibition towards various compounds. Forty fungal isolates were screened for ODC production, twenty fungal isolates have the higher potency to grow on L-ornithine as sole nitrogen source. *Aspergillus terreus* was the most potent ODC producer (2.1 µmol/mg/min), followed by *Penicillium crustosum* and *Fusarium fujikuori*. These isolates were molecularly identified based on their ITS sequences, which have been deposited in the NCBI database under accession numbers MH156195, MH155304 and MH152411, respectively. ODC was purified and characterized from *A. terreus* using SDS-PAGE, showing a whole molecule mass of ~110 kDa and a 50 kDa subunit structure revealing its homodimeric identity. The enzyme had a maximum activity at 37 °C, pH 7.4–7.8 and thermal stability for 20 h at 37 °C, and 90 days storage stability at 4 °C. *A. terreus* ODC had a maximum affinity (*K_m_*) for l-ornithine, l-lysine and l-arginine (0.95, 1.34 and 1.4 mM) and catalytic efficiency (*k_cat_*/*K_m_*) (4.6, 2.83, 2.46 × 10^−5^ mM^−1^·s^−1^). The enzyme activity was strongly inhibited by DFMO (0.02 µg/mL), curcumin (IC_50_ 0.04 µg/mL), propargylglycine (20.9 µg/mL) and hydroxylamine (32.9 µg/mL). These results emphasize the strong inhibitory effect of curcumin on ODC activity and subsequent polyamine synthesis. Further molecular dynamic studies to elucidate the mechanistics of ODC inhibition by curcumin are ongoing.

## 1. Introduction 

Ornithine decarboxylase (ODC, EC 4.1.1.17) is a pyridoxal-5′-phosphate-dependent enzyme, catalyzing the decarboxylation of L-ornithine to the diamine putrescine as the precursor of polyamine biosynthesis [1]. Polyamines are profoundly implicated in a myriad of cellular processes, including regulation of DNA replication, transcription, translation and cellular signaling in response to the environmental conditions [2]. Although polyamines with different cellular functions have been found in all organisms—bacteria, fungi, plants and human—the unique route for polyamine biosynthesis in fungi and animals is the *de novo* decarboxylation of ornithine into putrescine via ODC catalysis [3]. Putrescine is converted into spermidine by adding an aminopropyl group from the decarboxylated *S*-adenosylmethionine by spermidine synthase. Furthermore, aminopropyl group is added to spermidine by spermine synthase to form spermine [4]. Although spermine has not been reported in fungi, several orthologs of spermine synthase had been found in ascomycetous human fungal pathogens [5,6,7]. Recently, thermo-spermine, an isomer of spermine, was confirmed to be present in fungi [8,9].

The effectiveness of ODC in modulating polyamine synthesis in *Neurospora crassa*, *Schizosaccharomyces pombe* and *Saccharomyces cerevisiae* has been extensively studied [10,11,12]. Regulation of ODC activity by specific ODC antizyme for proteasome degradation is one of the unique mechanisms for modulation of polyamine synthesis in fungi [13,14] and human [12,15]. However, in fungi only a single antizyme has been reported, while in human, several antizyme- encoding genes were identified [16,17]. Polyamines play a major role in controlling cellular signal transduction, modulating protein-protein and protein-DNA interactions, as extensively documented in fungi [2]. In addition to the ODC-antizyme regulating mechanism, the ODC activity is highly regulated by growth stimuli and polyamines themselves [18]. Control of ODC post-translational modifications, without affecting its mRNA levels, to increase its catalytic efficiency is the most conspicuous feature of ODC regulation by polyamines [13]. 

In fungi, the regulatory mechanisms of ODC maturation were found to be consistent with those reported in mammalian systems [10]. Elevated levels of ODC expression have been observed in many human epithelial cancers such as colon, skin, and prostate [19], and this upregulation of ODC has been directly correlated to tumorigenesis and cellular proliferation [20,21]. ODC expression could be regulated by a number of factors such as hormones, tumor promoters and growth factors [22]. The higher antizyme expression to downregulate the activity of ODC, is one of the remarkable metabolic criteria demonstrating the functionality of antizymes as tumor suppressor proteins [23]. Thus, targeting the ODC activity to prevent polyamine synthesis is a promising approach for cancer therapy [24,25]. The potential anticancer activity of several ODC inhibitors such as difluoromethylornithine [26] and curcumin [27] has been recognized. Difluoromethylornithine (DFMO) is an irreversible suicide inhibitor of ornithine decarboxylase which is involved in polyamine synthesis. Polyamines are important for cell survival, thus DFMO was studied as an anticancer agent and as a chemopreventive agent [26]. Curcumin (diferuloylmethane) is a polyphenol derived from *Curcuma longa*, it has been used extensively in therapeutic applications for its antioxidant, analgesic, anti-inflammatory and antiseptic activity. Recently curcumin has been found to possess anticancer activities via its effect on a variety of biological pathways involved in oncogene expression, cell cycle regulation, apoptosis and tumorigenesis. Curcumin has shown an anti-proliferative effect against multiple cancers, via decreasing the ODC activity that is frequently upregulated in cancer and other rapidly proliferating tissues. Numerous studies have demonstrated that pretreatment with curcumin can impede carcinogen-induced ODC activity and tumor development in rodent tumorigenesis models targeting various organs, so the biochemical characterization of ODC from fungi as human ODC orthologs was the main objective of this study. Although the pivotal role of ODC in modulation of polyamine synthesis in fungi and human has been extensively studied, the biochemical properties and kinetics of inhibition of this enzyme from filamentous fungi remains ambiguous. Thus, the main objective of this work were: (1) To purify and molecularly characterize ODCs from various saprophytic and endophytic fungi, as a model enzyme with similar properties to human ODC and (2) To assess the kinetics of ODC inhibition by various compounds. 

## 2. Results and Discussion

### 2.1. Screening for the Potent ODC Producing Fungi 

Forty fungal isolates were grown on l-ornithine as sole nitrogen source and the developed fungal colonies were inspected. A plausible fluctuation of the assimilating ability of the tested fungi for l-ornithine as sole nitrogen source were observed (Table S1). Among these fungi, twenty isolates displayed the potency to utilize l-ornithine as sole nitrogen source revealing their possessing a highly active ODC, compared to the non-ornithine utilizing fungi (Table 1). From these potent ornithine utilizers, ten isolates were saprophytic and ten isolates were endophytes of *Podocarpus gracilior*. The productivity of ODC was assessed by growing the selected fungal isolates on liquid medium. The highest ODC productivity was assessed for *Aspergillus terreus* MS3 (0.286 µmol/mg/min) followed by *Penicillium crustosum* (0.229 µmol/mg/min), *Fusarium fujikuroi* (0.199 µmol/mg/min), *A. oryzae* (0.130 µmol/mg/min) and *A. terreus* PC (0.126 µmol/mg/min). While, the productivity of ODC by the other fungal isolates were ranged from 0.04–0.09 µmol/mg/min. The identification of the most potent fungal isolates producing ODC have been confirmed based on the molecular sequence of ITS regions. 

### 2.2. Molecular Confirmation of the Identity of the Potent ODC Producing Fungi

**Figure 1:**
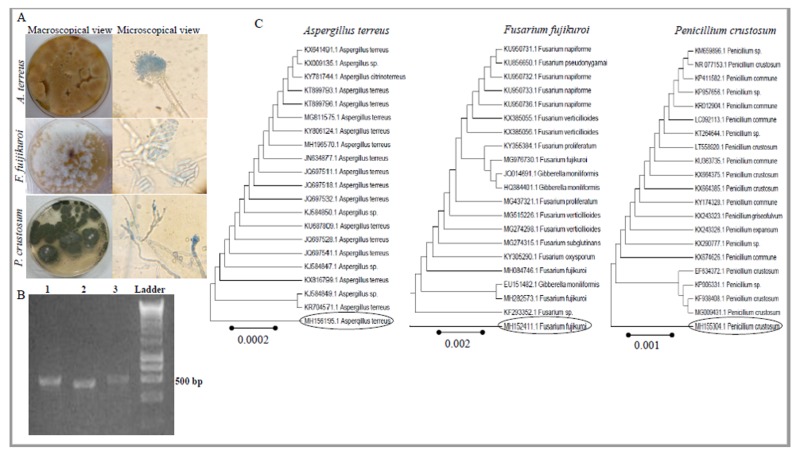
Morphological and molecular identification of the potent ODC-producing fungal isolates. The isolates were grown on PDA medium, incubated for 8 days at 30 °C, then photographed by the digital camera and examined by light microscope. (**A**) The macroscopical (left side) and microscopical (right side) views of *A. terreus*, *F. fuijikuroi* and *P. crustosum*. (**B**) The PCR amplicon of ITS region for *A. terreus* (lane 1), *F. fuijikuroi* (lane 2) and *P. crustosum* (lane 3), using the gDNA as PCR template, comparing to 1 kb ladder. (**C**) Molecular phylogenetic analyses of *A. terreus*, *F. fujikuroi* and *P. crustosum* constructed by the Maximum Likelihood model [28].

The morphological identification of the potent fungal isolates producing ODC as *Aspergillus terreus*, *Penicillium crustosum* and *Fusarium fujikuori*, was further confirmed based on their ITS region sequences. Using gDNA of these fungi as template for PCR, the size of PCR amplicons ranged from 550–600 bp for these fungal isolates (Figure 1). These amplicons were purified, sequenced and non-redundant BLAST searched in the NCBI database. These fungi have been confirmed as *Aspergillus terreus*, *Penicillium crustosum* and *Fusarium fujikuori* and their sequences were deposited in the NCBI database with accession numbers MH156195, MH155304 and MH152411, respectively. From the alignment, applying the Neighbor-Join and BioNJ algorithm with Maximum Composite Likelihood approach, the phylogenetic trees of these sequences have been constructed (Figure 1). The *A. terreus* MS3 isolate MH156195 displayed a 99% similarity with database deposited isolates of *A. terreus* KJ584849, KR704571, KX816799, KJ584847, JQ697541, JQ697528, KU687809 and KJ584850, with *E*-value zero and query coverage 98%. The isolate *Penicillium crustosum* MS1 MH155304 exhibited 98% similarity with database deposited isolates of *P. crustosum* EF634372, KP006331, KF938408, MG009431, KX674626, KX290777, KX243326 and KX 243323 with E-value zero and query coverage 96%. While, the *Fusarium fujikuori* MS2 MH152411 had a maximum identity (95%) with database deposited isolates of *F. fujikuori* MG976730, KF293352, EU151482, JQ014691, HQ384401, MH084746, KY305290 and MG2734315 with *E*-values zero and query coverage 97%. 

### 2.3. Nutritional Optimization of A. terreus for ODC Production 

**Figure 2:**
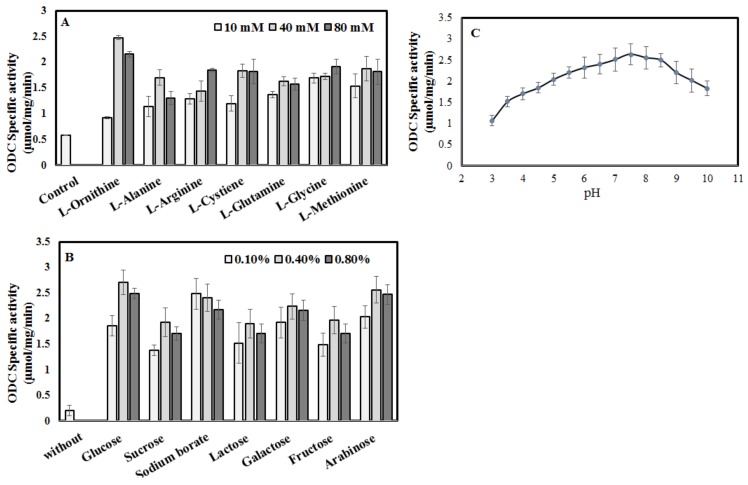
Cultural optimization of *Aspergillus terreus* to maximize its productivity for ODC. Different nitrogen sources (**A**), carbon sources (**B**) and initial pH values of the medium (**C**) were evaluated based on the ODC productivity by *A. terreus*.

The productivity of ODC by *A. terreus* in response to different carbon and nitrogen sources has been investigated. The basal medium was amended with various nitrogen sources such as l-ornithine, l-alanine, l-arginine, l-cysteine, l-glutamine, l-glycine and l-methionine at various concentrations (10, 40, 80 mM). Basal medium free of nitrogen was used as negative control. From the results (Figure 2), the ODC productivity by *A. terreus* has been maximally induced by about five-fold upon using l-ornithine as nitrogen source at 40 mM, compared to nitrogen-free medium. Other than l-ornithine, all the tested nitrogen sources have a relative similar induction effect on ODC productivity by *A. terreus*, suggesting the relative independence of ODC induction on the type of nitrogen source. Overall, the growth and ODC productivity of *A. terreus*, have been dramatically increased upon addition of all the tested nitrogen sources, proving the essentiality of nitrogen for regular fungal growth and metabolism. Similar results were reported for other PLP-dependent enzymes such as l-methionine γ-lyase, homocysteine γ-lyase and cystathionine γ-lyase from *A. flavipes*, *A. carneus* and *A. fumigatus* [29,30,31]. Consistent with our results, ODC expression in fungi was reported as a house-keeping enzyme that is independent on the microbial nutritional stress [32]. 

The impact of different carbon sources on growth and ODC induction has been assessed too. Various carbon sources, namely glucose, sucrose, lactose, galactose, fructose, arabinose and sodium borate with different concentration (0.1, 0.4 and 0.8%) have been amended into the basal carbon-free medium, then inoculated with fungal spores and incubated under standard conditions. Then the crude enzyme has been extracted and its activity and concentration determined.

The yield of ODC was dramatically increased upon addition of the different carbon sources, compared to the basal medium minus carbon (Figure 2). The highest activity of ODC (2.8 µmol/mg/min) was reported when using 0.4–0.8% glucose, followed by sodium borate, galactose, arabinose, fructose, lactose and sucrose. The yield of ODC has been increased by about 15-fold upon addition of glucose (0.4%) to the basal ODC production medium. The feasibility of rapid utilization of glucose as carbon source for the different metabolic pathways by the fungal cells has been documented extensively. The relative non-significant variations on the yield of ODC by *A. terreus* grown on different carbon sources confirms the constitutive production identity of this enzyme in fungi [32,33]. Thus, it could be concluded that the production of ODC by fungi is physiologically relatively independent of the identity of carbon and nitrogen sources in the production medium.

The induction of ODC by *A. terreus* in response to growing at different initial pHs (3.0–10.0) has been assessed. The maximum ODC activity has been detected by growing the fungus on modified glucose-ornithine medium with initial pH 6.0–8.0 (Figure 2). However, the fungal growth and ODC productivity have been strongly reduced upon growing under acidic conditions, compared to the little effect of slightly alkaline conditions. Similar results showing a higher negative effect of acidic conditions on fungal growth and their enzyme productivities, compared to a mild effect at higher pH alkaline conditions have been reported [29,34]. 

### 2.4. Purification, Molecular Mass and Subunit Structure of ODC from A. terreus 

*A. terreus* was grown in the optimized medium, and the crude ODC was extracted and purified by gel-filtration and ion-exchange chromatography [34,35,36]. By ion-exchange chromatography, the specific activity of ODC (1.6 µmol/mg/min) was increased by about 7-fold compared to the crude enzyme (0.23 µmol/mg/min) with 75.6% overall yield. The molecular homogeneity of the most active fractions was checked, and only the most homogenous fractions were gathered and concentrated prior to the next purification step. Upon using gel-filtration chromatography, the specific activity of ODC was increased by about 9-fold (2.1 µmol/mg/min) compared to the crude enzyme with about 50% overall yield. The overall purification profile of ODC from *A. terreus* has been summarized in Table 2. 

**Figure 3:**
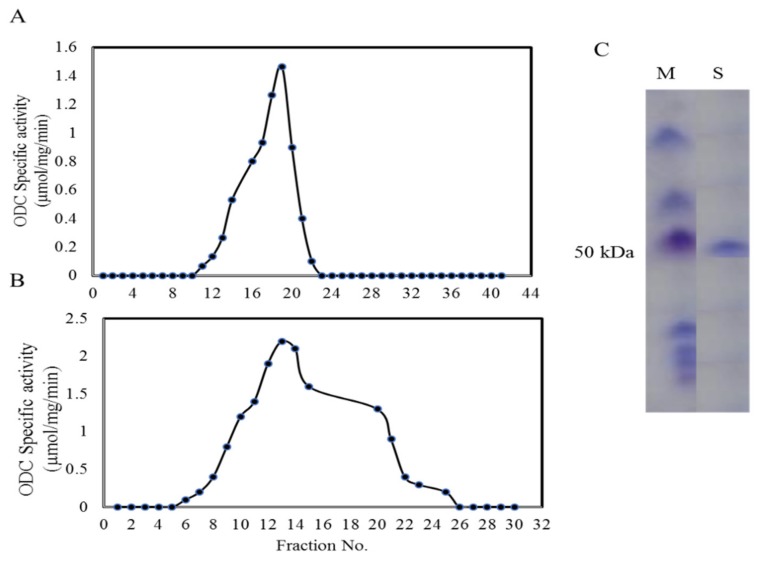
Purification of ODC from the culture of *A. terreus.* The crude ODC was extracted from the cultures of *A. terreus*, concentrated by dialyzer, purified by ion-exchange and gel-filtration chromatography. The pattern of ODC purification by ion-exchange chromatography (**A**), and gel-filtration chromatography (**B**). SDS-PAGE pattern for the subunit structure of purified *A. terreus* ODC (**C**). M, protein Marker (Cat. # PG-PMT2962, 315-10 kDa), S, purified ODC sample after the last purification column.

The homogeneity and overall purification steps were monitored by SDS-PAGE analysis (Figure 3). By the last purification step, a single proteineous band of molecular mass ~50 kDa was resolved, while with non-denaturing PAGE, the molecular mass of this band was close to 110 kDa, suggesting a homodimeric identity (two structural subunits identical in their molecular mass) of ODC from *A. terreus*. The molecular subunits and entire mass of *A. terreus* ODC was coincident with the molecular structures of ODC of *Candida albicans* [37], *Neurospora crassa* [18], *Saccharomyces cerevisiae* [38]. 

### 2.5. Biochemical Properties of A. terreus ODC 

#### 2.5.1. Reaction Temperature, Thermal Stability, Reaction pH and pH Stability

**Figure 4:**
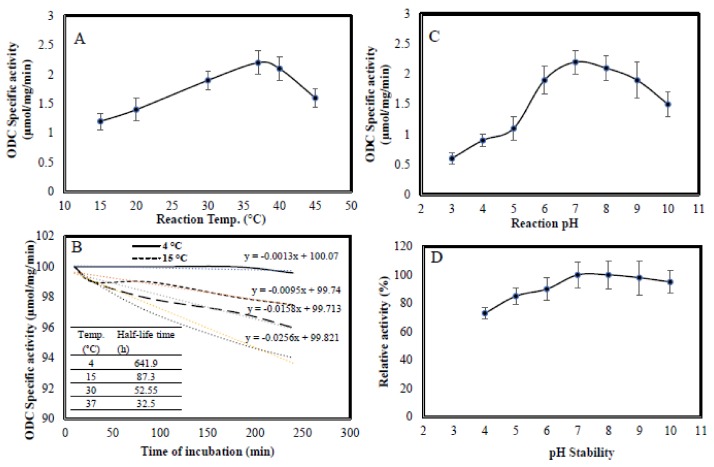
Biochemical properties of ODC purified from *A. terreus*. Reaction temperature (**A**), thermal stability (**B**), reaction pH (**C**) and pH stability (**D**).

The effect of reaction temperature on the activity of purified *A. terreus* ODC was assessed. From the results (Figure 4), the maximum ODC activity was measured at a reaction temperature of 36–39 °C, with a dramatic reduction of the enzyme activity being observed at 45 °C. However, at 20 °C, the activity of enzyme was reduced by about 50% compared to the control reaction at 37 °C. The maximum ODC activity at 37 °C could be explained by the highest activation energy to the substrate to bind with the enzyme as well as, the proper orientation of ODC to bind with substrate. The optimum activity of *A. terreus* ODC at 37 °C is similar to that of human ODC, suggesting their molecular and catalytic consistence [25]. 

The thermal stability of *A. terreus* ODC was investigated at different temperatures (4, 20, 37, 45 °C). From the thermal stability profile (Figure 4), the half-life time of ODC at 20, 37, and 45 °C was 90, 20 and 6 h, respectively. However, the enzyme has a relatively higher structural and catalytic stability by storage at 4 °C as revealed from the half-life time that estimated by about 120 days as calculated from the inference of the linear equation line (Figure 4). The loss on ODC activity could be due to the release of PLP coenzyme from the structural holoenzyme to form inactive apo-ODC, which is consistent with results reported for other PLP-dependent enzymes [39].

The effect of reaction mixture pH on the activity of *A. terreus* ODC was estimated. The pH was adjusted by citrate phosphate buffer and potassium phosphate buffer (ranged from pH 4–10) and the activity was measured by the standard assay. The highest activity for ODC was recorded at a reaction pH of 7.0–7.6 (Figure 4). Practically, for more acidic pH and alkaline pH, a reduction of the activity of ODC is reported. However, a strong reduction on the ODC activity was observed under acidic reaction conditions (pH 3.0–5.0), with a relatively mild reduction at alkaline pHs (pH 8–10). From the pH stability pattern (Figure 4), the enzyme displayed a higher stability at pH range 7.2–7.9, with dramatic reduction to its activity at acidic pHs and higher alkaline pHs. The strong loss on *A. terreus* ODC activity at acidic pHs, could be due to change on the ionic state of enzyme, causing dissociation of the subunits, and structural denaturation. Cleavage of the internal Schiff base of the PLP and lysine residue of Apo-ODC with storage at acidic and alkaline pH is the most recognizble feature for most of PLP-enzyme [29,34,40]. The precipitation pH of the *A. terreus* ODC was estimated by incubation of the enzyme at different pHs at 4°C overnight, then quantification of the precipitated ODC. From the results (data not shown), the enzyme was precipitated (*pI*) at pH 6.5–6.9, revealing the neutralization of total charge of the ODC at this pH range, similar results were observed for l-methioninase as PLP-dependent enzyme [34]. 

#### 2.5.2. Substrate Specificity and Kinetic Parameters 

The specificity of purified *A. terreus* ODC towards various amino acids as substrates has been evaluated based on the putative enzymatic byproducts such as ammonia, citrulline and putrescine (see Materials and Methods). Different amino acids were used as substrate for ODC under the standard assay normalizing to l-ornithine as standard substrate. The putative deaminating, deiminating and decarboxylating activity of ODC for each substrate was assessed (Table 3). From the results, purified *A. terreus* ODC displayed a reasonable activity towards l-arginine, l-lysine comparing to l-ornithine as standard substrate. On the contrary, the purified ODC had no detectable affinity towards any of the other tested amino acids such as l-methionine, l-glycine, l-tyrosine, l-valine, l-cysteine, l-alanine, l-asparagine, l-glutamine, and l-tryptophan in addition to *N*-acetylglucosamine, as revealed from Nessler’s, diacetylmonoxime and TNBS assays. By the standard assay for ODC based on released putrescine, the relative activity of ODC towards l-arginine and l-lysine is 16.2 and 20.9%, respectively, normalizing to l-ornithine as standard substrate. However, the enzyme displayed a higher deiminating activity towards l-arginine and l-lysine as substrates by 194 and 128%, respectively, compared to l-ornithine. The relative higher activity of ODC towards l-arginine and l-lysine could be due to their close stereo-structural similarity with the l-ornithine, having the same binding mechanism with the ODC active sites. Thus, the kinetic parameters for ODC catalytic activity towards l-ornithine, l-arginine and l-lysine have been determined. For l-arginine and l-lysine, the activity of ODC has been determined based on the released imino groups by diacetylmonoxime reagent [41], while the activity towards l-ornithine was determined by a standard TNBS assay [42]. Consistently, ODC from *Nicotiana glutinosa* had higher affinity towards l-ornithine, l-lysine and l-arginine [43].

The kinetic parameters of *A. terreus* ODC for these substrates are summarized in Table 4. *A. terreus* ODC displayed a maximum affinity and velocity towards l-ornithine (*K_m_* 0.9 mM, *V_max_* 4.8 µmol/mg/min), with relatively similar affinity and velocity for l-lysine and l-arginine (*K_m_* 1.3–1.4 mM, *V_max_* 4.1–3.8 µmol/mg/min) as substrates. Likewise, the highest turnover number (*K_cat_*) and catalytic efficiency (*K_cat_*/*K_m_*) for *A. terreus* ODC had been reported for L-ornithine (4.3 × 10^−5^·S^−1^ and 4.6 × 10^−5^ mM^−1^·S^−1^) followed by l-lysine (3.8 × 10^−5^ S^−1^ and 2.8 × 10^−5^ mM^−1^·S^−1^) and l-arginine, that in partially consistent with those kinetic parameters reported for ODC from *Nicotiana glutinosa* [43]. 

### 2.6. Effect of Inhibitors on Activity of Purified A. terreus ODC 

The amino acid identity of the active sites implicated in the catalysis of *A. terreus* ODC has been scrutinized by the response to suicide inhibitors, namely propargylgycine (PPG), guanidine thiocyanate, hydroxylamine, iodoacetamide, DTNB, MBTH and curcumin. The enzyme was incubated with each compound at concentrations of 5, 40, 200 and 400 µg/mL for 2 h, then amended with the l-ornithine, and their residual activity was measured. The inhibitory effect of these compounds on the ODC activity has been evaluated based on their IC_50_ as summarized in Table 5. The ODC activity was dramatically reduced upon using curcumin with IC_50_ 0.04 µg/mL. Consistently, curcumin has been extensively reported a strong inhibitor to the activity of tumor cells ODC, modulating the cellular polyamine metabolism [44]. Elevated polyamine synthesis was recognized as profound compounds for survival and rapid proliferation of tumor cells [45], thus, targeting the ODC, rate limiting enzyme of polyamine synthesis, has been considered as powerful chemopreventive therapy for tumor cells [46,47,48]. The IC_50_ of PPG for *A. terreus* ODC was 20.9 µg/mL, revealing the partial implication of l-cysteine in the catalytic process of ODC, since PLP has a higher reactivity to surface cysteinyl residues on the enzyme surface [39,49]. The enzyme irreversible inactivation by carbonyl reagents such as PPG, ensures the PLP dependence of these enzymes [50,51,52]. The activity of ODC was reduced by ~50% with addition of hydroxylamine (IC_50_ value 32.9 µg/mL) and MBTH (IC_50_ value 83.1 µg/mL), revealing the participation of surface lysine residues on the catalysis process of ODC, in which these carbonyl reagents cleavage the internal aldimine linkage between the PLP moieties and surface lysine residues [27,53]. The activity of *A. terreus* ODC was significantly reduced upon addition of iodoacetamide (IC_50_ 69.5 µg/mL) and DTNB (IC_50_ 83.6 µg/mL) revealing the implication of surface sulfur amino acids on the catalytic process and enzyme stability [49,54,55]. 

## 3. Materials and Methods 

### 3.1. Materials 

l-Ornithine, putrescine and pyridoxal 5-phosphate (PLP), polyacrylamide solution and 5,5-dithiobisnitrobenzoic acid (DTNB) were purchased from Sigma–Aldrich Co. (Saint Louis, MO, USA). 3-Methyl-2-benzothiazolinone hydrazone hydrochloride was obtained from Merck Co. (Darmstadt, Germany). All other chemicals were of analytical grade.

### 3.2. Screening for the Potent ODC Producing Fungal Isolates 

Forty fungal isolates were selected from our lab stock culture (EFBL, Faculty of Science, Zagazig University, Zagazig, Egypt) [29,30,56] and their capability to assimilate l-ornithine as sole nitrogen source was determined using modified Czapek’s-Dox agar media [57] containing 0.5% ornithine as nitrogen source were used. All the fungal isolates were grown on PDA for 6 days at 30 °C, a plug of each developed fungal isolate was centrally inoculated into the modified Czapek’s-Dox medium, incubated for 8 days at 30 °C. The developed fungal colonies were selected and screened for ODC production on the same liquid ornithine containing medium. A plug of the developed fungal isolate was inoculated into 50 mL/250 mL Erlenmeyer conical flaks, incubated at 30 °C for 10 days. The fungal mycelial pellets were collected, washed by sterile potassium phosphate (PP) buffer (pH 7.0, 50 mM). The fungal intracellular crude proteins were extracted by pulverizing their mycelial pellets (5 g fresh weight) in liquid nitrogen, dispensing in 10 mL PP buffer (pH 7.0, 50 mM) of 1 mM EDTA, 1 mM PMSF, 1 mM DTT, 10 µL 2-mercaptoethanol and 10 µM PLP [35,58]. The mixture was vortex for 5 min, then centrifuged at 8000 rpm for 10 min at 4 °C. The supernatant was used the crude source for ODC, the activity and concentration of ODC were determined. 

### 3.3. ODC Activity and Concentration 

The activity of ODC was determined based on the amount of released putrescine using 2,4,6-*tri*-nitrobenzene sulfonic acid (TNBS) reagent [42]. The reaction mixture containing 10 mM l-ornithine in PP buffer (pH 7.0), 20 µM PLP and 250 µL of crude enzyme in 1 mL volume was incubated at 37 °C for 30 min. The reaction was stopped by 10% TCA then neutralized by adding adequate amount of 2N NaOH. 1-Pentanol (2 mL) was added was added to the reaction mixture which was subsequently vortexed for 1 min and centrifuged at 5000 rpm for 5 min. The upper layer was transferred into a new tube, combined with an equal volume of 0.1 M sodium borate (pH 8.0). After vigorously mixing, 500 µL of TNBS (10 mM) reagent was added, subjected to 1 min vortex before adding 2 mL DMSO, and centrifuging at 6000 rpm for 5 min and left to separate into two layers. The upper layer containing TNP-putrescine-TNP was collected by micropipette and the absorbance at λ = 420 nm will reveal putrescine concentration based on a parallel measurement of authentic putrescine at the same conditions. The enzyme protein concentration was measured by Folinʹs reagent [59], comparing to a known concentration of bovine serum albumin.

### 3.4. Morphological and Molecular Identification of the Potent Fungal Isolates 

The potent fungal isolates producing ODC have been identified based on their morphological features according to the universal identification keys for the genera *Aspergillus* [60], *Penicillium* [61] and *Fusarium* [62]. The morphologically identified fungal isolates have been further confirmed based on the sequence analysis of the internal transcribed spacers (ITS) region (18S-ITS1-5.8S-ITS2-28S) [63] with minor modifications [30,56]. The fungal gDNA was extracted with cetyltrimethylammonium bromide (CTAB) reagent [64]. The fungal mycelia (0.2 g) were pulverized in liquid nitrogen then suspended in 1 mL CTAB extraction buffer (2% CTAB, 2% PVP40, 0.2% 2-mercaptoethanol, 20 mM EDTA, 1.4 M NaCl in 100 mM Tris-HCl (pH 8.0)). The fungal gDNA was used as template for PCR with primers; ITS4 5′-GGAAGTAAAAGTCGTAACAAGG-3′ and ITS5 5′-TCCTCCGCTTATT GATATGC-3′ using 2× PCR master mixture (*i*-Taq^TM^ Cat. # 25027, INTRON Biotechnology, Jungwong-gu, South Korea) according to the manufacturer’s instruction. The PCR amplicons were resolved on 1.5% agarose gel in 1× TBE buffer (Cat. # AM9864, Ambion, Invitrogen™, Carlsbad, CA, USA), normalizing to 1 kb DNA ladder (Next-gene ladder, Cat. # PG10-55D1, Puregene) visualized by gel documentation system. The amplicons were eluted and sequenced (Applied Biosystems Sequencer, HiSQV Bases, Version 6. (Foster, CA, USA)) using the same primer sets. The retrieved sequences were BLAST searched with non-redundant sequences on the NCBI database. For the multiple sequence alignment, the FASTA sequences were imported into MEGA 7.0 portal aligned with ClustalW muscle algorithm [65], the phylogenetic tree was constructed using the neighbor-joining method [28]. 

### 3.5. Purification, Homogeneity and Molecular Subunit Structure of Aspergillus terreus ODC 

Based on its ODC activity, *Aspergillus terreus* was selected as the potent ODC producer. *A. terreus* was grown on the nutritionally optimized medium as follow; 0.2% ornithine, 0.4% glucose, 0.1% KH_2_PO_4_, 0.05 % MgSO_4_. 5 H_2_O and 0.05% KCl in distilled water, and the medium pH was adjusted to 7.0. One mL of *A. terreus* spores (10^5^ spore/mL) were inoculated to 50 medium/250 mL Erlenmeyer conical flask, incubated at 30 °C for 8 days, under 100 rpm shaking. The mycelial pellets were collected, washed by sterile potassium phosphate buffer. The fungal pellets (100 g) were pulverized in liquid nitrogen, dispensed in 100 mL extraction buffer PP with 1 mM PMSF, 1 mM EDTA and 10 µM PLP. The fungal intracellular proteins were extracted as described above. 

The enzymatic preparation was fractionally concentrated with 20 kDa cut-off dialyzer (20 kDa, 546-00051 Wako Chemicals, USA) against the same buffer, then concentrated by dialysis against polyethylene glycol 6000 [49,66]. The concentrated ODC was purified by ion-exchange chromatography using a DEAE-Sepharose column (1.5 × 40 cm). After loading of the sample and pre-equilibration of column by PP buffer, the enzyme was eluted with the same buffer of gradient NaCl (50–200 mM) at 1 mL/min flow rate. The activity and concentration of ODC were determined for each fraction as described above. The molecular homogeneity of the active fractions was assessed by SDS-PAGE, the most active and molecular homogenous fractions were gathered, downstream purified by gel-filtration chromatography by Sephadex G100 column [67]. The enzyme was loaded into the pre-equilibrated column, and eluted by PP buffer containing 10 µM PLP. The activity and concentration of ODC were determined as described above. The molecular homogeneity was assessed by SDS-PAGE, the most active and homogenous ODC fractions were collected and concentrated with 10 K ultra-centrifugal membrane and stored at 4 °C for further analysis. 

The molecular homogeneity and subunit structure of purified *A. terreus* ODC were checked by SDS-PAGE [68] with slight modifications [49]. The molecular mass of the ODC subunit were determined, normalizing to authentic protein marker (Puregene, 3 color Broad Range Cat. # PG-PMT2962 315-10 kDa). The entire molecular mass of the purified ODC was determined by native-PAGE [49]. 

### 3.6. Biochemical Properties of the Purified A. terreus ODC 

The optimum reaction temperature for ODC activity was determined by incubating the reaction mixture at different temperatures (30–45 °C), then measuring the activity of ODC by a standard assay. The thermal stability of purified *A. terreus* ODC was evaluated by preincubation of the enzyme at different temperatures (30–45 °C), then measuring the residual activity after 30, 60, 180 and 240 min by standard assay. The thermal kinetic parameters such as half-life time (*T_1/2_*), and thermal inactivation rate (*kr*) were determined [36,66]. 

The storage stability of the enzyme at 4 °C was determined. The enzyme was stored at 4 °C, then its residual activity was measured after 5, 10, 20, 30, 60 and 90 days by the standard assay. 

The optimum pH for the purified *A. terreus* ODC was assessed by adjusting the reaction mixture at different pHs (3–10), then measuring the activity of ODC by a standard assay. The pH stability of purified ODC was evaluated by pre-incubation of the enzyme at different pHs (5.0–9.0) using PP buffer, the enzyme preparation was kept at 4 °C for 2 h, then residual activity of ODC was measured by the standard assay. 

The affinity of ODC towards different amino acids such as l-ornithine, l-arginine, l-lysine, *N*-acetylglucosamine, l-methionine, glycine, tyrosine, valine, l-cysteine, l-alanine, l-glutamine, l-asparagine was evaluated by the same reaction assay. The putative enzymatic byproducts such as ammonia, citrulline and putrescine were determined by Nessler’s reagent [66], diacetylmonoxime reagent [36] and TNBS [42], respectively, under standard conditions. The kinetic parameters of *A. terreus* ODC; Michalis-Menten constant (*K_m_*), maximum velocity (*V_max_*), and turnover number (*k_cat_*) and catalytic efficiency towards the most hydrolysable amino acids were calculated. 

### 3.7. Effect of Various Inhibitors on ODC Activity

Active site prediction using amino acids analogues has been recognized as one of the mechanisms for interrogation of enzymatic identity [39]. The influence of various inhibitors namely 5,5′-dithiobis-2-nitrobenzoic acid, 3-methyl-2-benzothiazolinone-hydrazone, guanidine thiocyanate, iodoacetamide, hydroxylamine, propargylglycine and curcumin on the activity of purified ODC was estimated. The enzyme was incubated with different inhibitor concentrations (5, 40, 200, 400 µg/mL) for 2 h at 30 °C, then amended with the substrate, and the residual activity of the enzyme were determined by the standard assay. The inhibitory kinetic parameters were measured according to El-Sayed et al. [36]

### 3.8. Deposition of the Fungal Isolates 

The potent isolates producing ODC, *Aspergillus terreus*, *Penicillium crustosum* and *Fusarium fujikuroi* have been deposited into the NCBI database under accession numbers MH156195, MH155304 and MH152411, respectively. 

### 3.9. Statistical Analyses

All experiments were conducted in biological triplicates, the results were expressed by means ± STDEV. The significance and *F*-test were calculated using one-way ANOVA with Fisher’s Least Significant Difference post hoc test. 

## 4. Conclusions

In conclusion, ODC was screened from various saprophytic and endophytic fungi, *A. terreus* MS3 displayed the highest potentiality to utilize l-ornithine as sole nitrogen source for production of ODC. The optimization processes revealed the partial independence of ODC expression on the type of medium carbon and nitrogen source. The enzyme was purified from *A. terreus* and its biochemical and catalytic properties were assessed. Since targeting of human tumor cells ODC is currently a promising therapy for different tumor types, thus, searching for a novel/ efficient compound to target this enzyme was the ultimate objective of this work. Due to the identical biochemical structure of the fungal and human ODCs, fungal enzyme was used as a model on this study. The activity of *A. terreus* ODC was completely inhibited by curcumin and DFMO with IC_50_ values ~0.04 µg/mL and 0.02 µg/mL, respectively. This study has emphasized on the inhibitory role of curcumin to the ODC functionality, and further molecular dynamic studies are ongoing to explore the mechanism of interaction of curcumin with the active sites and PLP-binding domains of ODC. 

## Figures and Tables

**Table 1:** Activity of ornithine decarboxylase from the selected saprophytic and endophytic fungal isolates.

	No.	Fungal Isolates	Specific Activity (µmol/mg/min)
Saprophytic fungi	1	*Fusarium* sp	0.053
	2	*Aspergillus fumigatus*	0.053
	3	*Aspergillus flavus*	0.067
	4	*Aspergillus parasiticus*	0.079
	5	*Fusarium fujikuori*	0.199
	6	*Aspergillus terreus* MS3	0.286
	7	*Aspergillus oryzae*	0.130
	8	*Penicillium crustosum*	0.229
	9	*Aspergillus* sp1	0.074
	10	*Aspergillus* sp2	0.078
Endophytic fungi	11	*Aspergillus terreus* PC	0.126
	12	*Aspergillus flavus* PS1	0.059
	13	*Aspergillus versicolor*	0.049
	14	*Aspergillus flavus* PS2	0.049
	15	*Fusarium proliferatum*	0.064
	16	*Fusarium* PL1	0.043
	17	*Penicillium chermesinum*	0.048
	18	*Penicillium chrysogenum*	0.043
	19	*Fusarium* PL2	0.047
	20	*Aspergillus terreus* PS	0.042

**Table 2:** Overall purification profile of ODC from *A. terreus*.

Step	Total Activity (µmol)	Total Protein (mg)	Specific Activity (µmol/mg/min)	Purification Fold	Yield (%)
Crude enzyme	11757	51100	0.23	1	100
After 20 kDa cut-off dialyzer	8895.6	8640	1.17	5	75.6
Ion-exchange chromatography	7366.2	5709.4	1.6	7	62.8
Gel-filtration chromatography	5836.8	2778.8	2.1	9	50

**Table 3:** Substrate specificity of the purified ODC of *A. terreus.*

	Nessler’s Assay		Diacetyl Monoxime Assay		TNBS Assay	
	Activity (µmol/mg/min)	Relative Activity (%)	Activity (µmol/mg/min)	Relative Activity (%)	Activity (µmol/mg/min)	Relative Activity (%)
l-Ornithine	1.87	100	0.0035	100	2.1	100
l-Arginine	0.35	19	0.0068	194	0.34	16.2
l-Lysine	0	0	0.0045	128	0.44	20.9

**Table 4:** Kinetic properties of *A. terreus* ODC for l-ornithine, l-lysine and l-arginine.

Substrate	*K_m_* (mM)	*V_max_* (µmol/mg/min)	*K_cat_* (s^−1^)	*K_cat_*/*K_m_* (mM^−1^·s^−1^)
l-Ornithine	0.95	4.8	4.3 × 10^−5^	4.61 × 10^−5^
l-Lysine	1.34	4.18	3.8 × 10^−5^	2.83 × 10^−5^
l-Arginine	1.4	3.8	3.4 × 10^−5^	2.46 × 10^−5^

**Table 5:** Effect of different amino acids suicide analogues on the activity of *A. terreus* ODC.

	Concentration (µg/mL)	Relative Activity (%)	IC_50_ (µg/mL)
Control	0	100	-
Propargylgycine	5	89	20.9
	40	30	
	200	17	
	400	6	
Guanidine thiocyanate	5	90	210.9
	40	60	
	200	30	
	400	37	
Hydroxylamine	5	45	32.9
	40	13	
	200	0	
	400	0	
Iodoacetamide	5	37	69.5
	40	19	
	200	10	
	400	0	
5-5′-Dithiobis-(2-nitro-benzoic acid)	5	39	83.6
	40	27	
	200	14	
	400	5.8	
3-Methyl-2-benzo-thiazolinone hydrazone	5	41	83.1
	40	29	
	200	12	
	400	0	
Curcumin	5	11	0.04
	40	0	
	200	0	
	400	0	
DFMO	5	8	0.02
	40	0.1	
	200	0	
	400	0	

## Supplementary Materials

The following are available online, Table S1: Potentiality of recovered fungal isolates to grow on modified Czapek’s-Dox medium of L-ornithine as sole nitrogen source.
