# Supplementary material for: Purification and Characterization of Ornithine Decarboxylase from Aspergillus terreus; Kinetics of Inhibition by Various Inhibitors
Source: Molecules. 2019 Jul 29;24(15):2756. doi: 10.3390/molecules24152756 (PMC6696095; doi:10.3390/molecules24152756)
Supplement: Supplementary file 1 [file molecules-24-02756-s001.pdf]

Table S1: Potentiality of recovered fungal isolates to grow on modified Czapek's-Dox medium of L-ornithine as sole nitrogen source.

Source	Isolate No.	Fungal isolate	Visual fungal growth on L-ornithine as sole N source
**Saprophytic fungi**	1	*Aspergillus terreus*	+++
	2	*A. fumigates*	+
	3	*A. flavus*	+
	4	*A. parasiticus*	++
	5	*A. oryzae*	+++
	6	*A. niger*	+
	7	*A. tamarii*	++
	8	*A. carneus*	-
	9	*A.sparsus*	-
	10	*A. niveus*	-
	11	*A.ochraceos*	-
	12	*A. flavipes*	-
	13	*A.candidus*	-
	14	*Fusarium* sp	+
	15	*F. fujikuroi*	+++
	16	*F. oxyporum*	-
	17	*Penicillium crustosum*	+++
	18	*P. notatum*	-
	19	*Humicola* sp	-
	20	*Colletotricum* sp	-
**Endophytic fungi**	21	*A. terreus* P.C.	++
	22	*A.flavus* P.S.1	+
	23	*A.flavus* P.S.2	+
	24	*A. versicolor*	+
	25	*A. terreus* P.S.	+
	26	*A.s fumigatus*	-
	27	*A. oryzae*	-
	28	*A. niger*	-
	29	*A. flavus var columnaris*1	-
	30	*A.flavus* P.S.3	-
	31	*A. flavus var columnaris*2	-
	32	*F.proliferatum*	+
	33	*Fusarium moniliforme*	+
	34	*Fusarium oxysporum*	+
	35	*P.chermesinum*	+
	36	*P.chrysogenum*	+
	37	*Cladosporium* sp	-
	38	*Cunninghamella* sp	-
	39	*Penicillium* sp P.L.	-
	40	*Penicillium* sp P.S.	-

Podocarpus gracilior cork (PC); Podocarpus stem (PS); Podocarpus leaves (PL).
